# Ethical implications of COVID-19 management—is freedom a desired aim, or a desired means to an end?

**DOI:** 10.3389/fpubh.2024.1377543

**Published:** 2024-04-26

**Authors:** Andro Košec, Filip Hergešić, Boris Zdilar, Lucija Svetina, Marko Ćurković

**Affiliations:** ^1^School of Medicine, University of Zagreb, Zagreb, Croatia; ^2^Department of Otorhinolaryngology and Head and Neck Surgery, University Hospital Center Sestre Milosrdnice, Zagreb, Croatia; ^3^Department of Otorhinolaryngology and Head and Neck Surgery, University Hospital Sveti Duh, Zagreb, Croatia; ^4^Department of Abdominal Surgery, University Hospital Sveti Duh, Zagreb, Croatia; ^5^Department of Cardiac Surgery, University Hospital Center Zagreb, Zagreb, Croatia; ^6^University Psychiatric Hospital Vrapče, Zagreb, Croatia

**Keywords:** ethics, COVID-19, vaccination, freedom, management

## Abstract

Most developed societies managed, due to their prosperity and resource abundance, to structure relationships among free individuals in such a way to leave them fundamentally unstructured, according to the free market principle. As the pandemic illustrated well, this lack of structure when facing collective threats makes it impossible to collectively and proportionately assess and manage its implications and consequences. This may be particularly precarious when introducing comprehensive, monitoring and tracking, surveillance systems dependent on the vaccination status of the individual. If our previously shared aims were successfully and collectively enacted with the greatest of costs, is it permissible that the degree of personal freedom is a commodity, and everyone is a compulsory participant? The need to control one’s COVID-19 status allows the individual to become legally free from excessive enactment of sovereignty of the state. Should these rights be regulated by the free market?

## Introduction

Testing, tracing, isolation and vaccination are critical disease transmission control measures. Among those, testing and vaccination are the ones that have most critically affected by our abilities to translate scientific knowledge into practice (1). However, testing availability, accuracy and utility remains limited, while vaccine effectiveness is burdened by possibility of ineffectiveness and timely distribution issues (2). In the acute COVID-19 pandemic setting, these limitations were one of main reasons behind the introduction of all-encompassing highly restrictive public health measures and their developing devastating consequences (3). In this scenario, everyone, weather voluntary or not, took their share of risks and rewards, based on a universal personal moral responsibility narrative (4). Societies struggled to tackle the immediate consequences emerging from the infectious threat creating a unique setting where rationing and prioritization of scarce health care resources were inevitable, and after the initial phase, shifting toward constraining or encouraging particular behaviors. Consequently, various guidelines were proposed trying to establish coherent (re)allocation of resources upholding basic ethical principles, such as justice, beneficence, non-maleficence and transparency. Although of greatest importance in pre-pandemic circumstances, the principle of autonomy (weather those of person in need or caregivers) is at greatest risk during this pandemic. Weather rightful or not, prioritization strategies occurring in the acute COVID-19 setting mirrored utilitarian principles, while the most beneficial aims of maximization were relatively easy to define and empirically measure – saving lives (4, 5). After four pandemic waves, there is a continuing need to be aware of a present infectious threat, and uphold some of the measures previously applied.

Underlying principles and strategies informing these processes should, at least in theory, reflect the societies’ most fundamental, valuable and worthy ethical principles. In turn, the main ethical issues during chronic pandemic management relate to a current and uneasy reexamination of the most fundamental values and principles within our society. It is by now painfully obvious that there are not many things that most of global societies could unanimously agree on, even the importance of fundamental human rights, personal autonomy and personal possession being in peril.

## Perspective

Human rights are about being free from any kind of coercion and being entitled to achieve one’s own desired life goals. In that sense, the need to constantly monitor one’s COVID-19 status impacts not only the ability to achieve future goals in life, but also the possibility of the individual to become legally free from the sovereignty of the state. As such, it has far-reaching direct and indirect consequences for the individual and society. Should these rights be regulated by the free market or should the state make unprecedented intervention once again and undermine the fundamental pillars of free market, this time erring on the side of freedom? The answer to this question is not easy, but it has to do a lot on what do we consider as most fundamental values, and finally, on how we perceive our freedom; as a desired aim, or a desired means to an end.

Most developed societies managed, due to their prosperity and resource abundance, to structure relationships among free individuals in such a way to leave them fundamentally unstructured, according to the free market principle. However, as the pandemic illustrated well, this lack of structure when facing collective threats makes it impossible to collectively and proportionately assess and manage its implications and consequences. Even if nearly everyone agrees that the threat is genuinely serious, many might have a substantially different idea of what survival, individually and collectively, actually means. In that sense, some people are actually more endangered by the infection, and some by the responses applied, especially when vulnerable populations are concerned. It is now clear that the impact of COVID-19 has led to higher infection and mortality rates in older adults and premorbid individuals, people with lower income and immunocompromised status, predisposing these groups to higher risk than the rest of the population (6, 7). Due to this increase of risk, erring on the side of security versus human rights can appear justified, but it nonetheless represents an unprecedented, and supposedly temporary intrusion of the state into the citizens’ lives and rights (6).

However, data from the last four years have shown that the COVID-19 pandemic and the responses to it have worsened health care access disparity, increased health communication challenges, worsened mental health and wellbeing, led to profound social and economic consequences and vaccination inequities (7).

If our previously shared aims to protect from unknown harm were successfully and collectively enacted with the greatest of costs, is it permissible that parameters of personal freedom are altered in a situation where everyone is a compulsory participant?

This may be particularly precarious when introducing comprehensive, monitoring and tracking, surveillance systems dependent on the vaccination status of the individual (6). Published literature on non-pharmaceutical interventions’ (NPI) impact show that respiratory hygiene had the highest compliance, above 41% whereas hand hygiene showed the lowest (4%), with significant differences between gender and place of residence (large cities versus regional cities) (7–9). Monitoring the results of inter-city traffic controls, restrictions of personal movement, along with self-isolation has shown a drop in measles (90%) and scarlet fever (95%) infection rates. The effect was less obvious, but noticeable in tuberculosis (19.6%), pertussis (76.5%), influenza (22%), and mumps (52.1%) infection rates in China, measured at usual peak incidence periods of the year. At the same time, seasonal peaks in the incidence of these respiratory infectious diseases disappeared in 2020 and 2021 (7). On the other hand, during the past two years of returning to normal, we witnessed an increase in the number of patients infected with common respiratory viruses, including RSV and human parainfluenza viruses that develop more severe diseases. A reduction of exposure to infectious agents resulting due to NPIs may have led to a less potent trained immunity in children, and a drop in heterologous protection against infections resulting in greater overall susceptibility to infections in the future (8, 9).

An acutely threatening COVID-19 setting triggered urgent, basic survival-oriented collective responses, enacted mostly through public institutions. Such responses were, and still are, guided predominantly by a utilitarian logic which allows maximization of benefits and relatively quick recalibration of responses in accordance to a rapidly changing context in a scenario where immunity within the population is low and pharmaceutical interventions are absent, which is representative of a typical situation during the (re-)emergence of infectious diseases for which therapeutic drugs or vaccines are not yet available (10). Since no single NPI is effective in controlling COVID-19 spread, curfews, lockdowns, as well as restricting locations for public gatherings, were the most effective NPIs reported in large studies (10, 11). Other studies have also assessed the efficacy of different NPIs related to case identification, environmental measures, healthcare, public health capacity, resource allocation, risk communication, social distancing, travel restriction, and returning to normal life, demonstrating that risk communication had the greatest impact on the population, especially highlighting the importance of efficient communication during a crisis (11, 12). Risk communication addressed and educated the population on hazards, exposures, vulnerability and disease control, showing that psychology-driven positive and negative feedbacks generate opposing behavior in large populations. The responses and scale of communication were also characterized by an unprecedented intrusion of the state in almost all social structures, followed by a growing backlash of citizens whose livelihood and basic freedoms are often curtailed temporarily, or permanently. However unprecedented, these interventions seemed necessary as the fundamental role of the state is to protect its citizens. The portfolio of initial interventions relied on environmental health and risk information quality, that were considered to plan primary non-coercive interventions at the population scale. The systemic evaluation of the effectiveness of interventions based on this experience is valuable for public health authorities to develop preparedness plans timely, evaluate interventions over time, and design policies to decrease population vulnerabilities in long term, culminating with the arrival of an effective vaccine (12, 13). Introduction of massive tracing, identification and quarantine strategies implied that the effectiveness for this intervention is without dispute, but was not seen in Japan, while present for other countries, such as USA and Italy. Case identification and contact tracing effectiveness was negative for European countries with elevated incidence such as France and Germany, as well as Asian countries such as India and South Korea, but the latter, after being praised as a great example of successful contact tracing was hit by a second pandemic wave that eliminated effective tracing (10–12).

As soon as the proposed vaccination rate had been identified in a certain society, immediate actions were taken by the lawmakers and health policy makers to enable, promote and ensure complete vaccination and, hopefully, disease control. However, after an initial upsurge of optimism and collective euphoria, a significant percentage of citizens chose not to vaccinate themselves and their underage children, due to heterogeneous issues, but still making a presumed 85% population vaccination rate hard to achieve. This was met by public vaccination campaigns and indirect forms of promoting vaccination, such as “covid-passports” and obligatory testing for all citizens using public institutions, and even by obligatory vaccination policies in some countries (7, 13). In order to ensure a core governance model acceptable to the public while establishing a private-public vaccination program, the guiding principles should be as simple as possible, transparent, and acceptable to all partners. The five key governance structures proposed in a successful vaccination program should involve a decision-maker or steering committee, a scientific committee, quality control and audit committee, implementer and a financial administrator, all subject to transparent decision-making rules and conflict of interest management to ensure maximum public trust (14).

It is clear that human behavior based on trust in authority plays an important role on the efforts to control the transmission of COVID-19, since the effectiveness of mitigation measures depends on NPI compliance and vaccine acceptance. Specifically, humans adopt protective behavior when social distancing measures are in effect, typically concurrent with a high number of infections, and thereafter reduce protective behavior when vaccination coverage is high or when mandated contact reduction measures are relaxed, typically concurrent with a reduction of infections (15).

These changes in behavior revealed and magnified pre-existing inequities and inequalities within the society as well as their profound and devastating consequences, for certain individuals and society as a whole. Here it is useful to invoke the metaphor used by Jock Young of “*actuarial cordon sanitaire*” one which separates the worlds of losers from that of the winners. Indeed, it seems that infective threat and our responses to it cumulatively contributed to such a separation. It is evident that many of these inequalities and inequities as well as their consequences must be discussed publicly, including the scientific community, but that the general public is also very keen on involving themselves in the scientific debate, causing further chaos, with mainstream and social media acting as catalysts (16). Nonetheless, general consensus and understanding on how we should proceed with pandemic management is crucial in ending this war of attrition between the state and the individual.

Both at legal and ethical levels, decision-making in public health during a pandemic should respect the non-derogable guidelines of fundamental human rights. However, the collision of fundamental rights represents a significant problem for government and healthcare management due to extension of the acceptable exercise of freedom rights in times of a pandemic (17, 18). Therefore, the pandemic has highlighted the impossibility to raise morality to the level of universality, creating an essential distinction between the subject of morality and the object of morality. Freedom is the possibility to be morally responsible of one’s acts. Unfree individuals are neither members nor active subjects of the moral sphere, since they have no moral relationships.

The contemporary debate on freedom in a case of a threat to human survival reflects a great expansion of our moral sphere. However, moral agents within this expanding moral sphere are equal only in principle. If anything, the pandemic has shown that freedom is obviously a matter of degree. This can be well illustrated in quite obvious fact that those parts of society that generally have less freedom are the ones that are most willing to fight for it. It is easy to bring to mind the riots or protests against COVID-19 related restrictions that are being silenced by those that are just above in the freedom chain. Freedom gradients is what keeps societies, as current order of things and beings, together.

When discussing those issues, it is useful to evoke a distinction between absolute and relative ethics, or as Erich Fromm formulated, universal and socially immanent ethics. Universal ethics aims regulating or supporting “growth and unfolding” of humans. On the other hand, socially immanent ethics constructs specific norms necessary for the functioning and survival of specific kind of society. In an acutely threatening setting, such as one created by SARS-CoV-2, it became evident that socially immanent ethics are primary in pandemic management, as survival depends on it (8, 9). However, socially immanent ethics, driven predominantly by immediate context, creates norms that are specific to certain groups, while significantly differing among different societies. Ethical dilemmas were aggravated during the COVID-19 pandemic, resulting in moral distress and eventually illness and job resignation. Compromising care due to structural constraints indicate the negative consequences of such unresolved dilemmas at the health system level and the inherent risk for patients’ health and wellbeing (18). In order to tackle the ethical dilemmas while maintaining effective pandemic management, several key areas need to be discussed; the identification and acceptance of human vulnerability; the discovery of positive paradigms in traumatic situations in society; the prevalence of the common good over the particular interest, as the core structure of any society. Healthcare benevolence is a necessary dimension of health care contrasted with global vulnerability, forming a new ethical landscape that ensures a humanistic curriculum in the training of all healthcare professionals (19, 20).

When discussing shared decision making, a management paradigm empowering patients as partners is necessary. The pandemic has altered healthcare delivery, but prompted re-evaluation of common practices and enhance effectiveness of management strategies. Navigating the uncertainty of subsequent pandemic waves creates confusion about how to safely recalibrate clinical service (20, 21). In general, patient participation has focused on short-term outcomes such as patient satisfaction, short-term clinical outcomes, or decisional conflict. However, the COVID-19 pandemic has elevated public participation in decision-making processes to a broader interactional/adaptable and organizational framework, where the public may contribute to a trade-off benefits versus harms and assess their burdens–in short, to new social norms in the public health and clinical setting (21, 22).

Where the international judicial system was concerned with pandemic management, it clearly shows that fundamental rights are essentially relative in the sense that there is no fundamental right, based on a principle, of absolute nature (19). When two principles collide, e.g., the principle of individual liberty and the principle of public health, deciding must be in favor of the fairest decision to take. The right to health in the context of the current pandemic must prevail over the right to unrestricted liberty of movement of people, because the health right in this pandemic carries more legal and moral weight than the liberty exercise with some necessary restrictions, considering also that the liberty exercise can never be without any restriction, for the common good of all people, creating a necessary legal norm (19, 20).

Norms necessary for survival are in conflict with universal norms that are necessary for full growth and development. The conflict between these two ethics has been lowered thought the process of evolution, but it will exist as long as interests of societies are not equal to interest of all its members.

Historically conditioned social necessities are being confronted with the existential need of individuals. Norms are always imposed through power and commercial interest. This plays out in such a way that societal institutions promote the interest of those groups whose bargaining power is so great that they can negotiate new rules and have power over their interpretation (22, 23). The ongoing strife between the need to enforce safety and safeguard freedom severely impacts the tendency to represent society without fundamental ethical contradictions. In addition, the discussion raises important questions on whether these norms are equally binding to all members of society.

Modern societies do not show a growing trend toward universal moral norms, but rather toward privatization, erosion of the state’s role in economic redistribution, and political abandonment of the state as a tool of major social transformation aimed at rectifying injustices and improving lives, exactly contrasting the major issues to improve public policy during the pandemic (23, 24). The increase of freedom can also be viewed as a circumscribed role of the state – apparent in governmental failures in creating comprehensive national platforms of non-pharmaceutical interventions and a heavy reliance on the private sector for functions ranging from public policy development to vaccine distribution (24). The model of public health research reliant on scientific investigation only has sidelined social needs and separated researchers and those working in public agencies. This approach often leaves public health officials in a weak position: left to rely on diplomacy, rather than law, to encourage action (24).

Our position in the freedom chain may well depend on, “rhetorical strategies of persuasion, and nothing else, as the bases for human moral codes.” (23, 24) Power, interest and corresponding privilege creates blind spots, as those agents with power to define freedom do not recognize their own privileges and tend to deny the resulting advantages. Every individual must be wary of the inherent tendency of the powerful to conserve the existing status quo that provides privilege, and especially so when faced with threats to it.

## Author’s note

This manuscript was assembled based on professional, academic and research opinions by specialists in public health, each from their own perspective; HF is a young medical doctor and researcher interested in scientific work, BZ is a military leader and public health manager. LS is a cardiac-surgeon with a MHA in health leadership, AK and MĆ are a well-versed duo of a psychiatrist and bioethicist and a head and neck surgeon and deontologist with a number of papers published on COVID-19, and are the guarantors of the article.

## Data availability statement

The original contributions presented in the study are included in the article/supplementary material, further inquiries can be directed to the corresponding author.

## Author contributions

AK: Conceptualization, Data curation, Formal analysis, Investigation, Project administration, Supervision, Validation, Visualization, Writing – original draft, Writing – review & editing. FH: Writing – original draft, Writing – review & editing. BZ: Writing – original draft, Writing – review & editing. LS: Writing – original draft, Writing – review & editing. MĆ: Conceptualization, Data curation, Formal analysis, Investigation, Methodology, Writing – original draft, Writing – review & editing.
